# Nitrogen, phosphorus, and potassium fertilization to achieve expected yield and improve yield components of mung bean

**DOI:** 10.1371/journal.pone.0206285

**Published:** 2018-10-25

**Authors:** Zhichao Yin, Wenyun Guo, Huanyu Xiao, Jie Liang, Xiyu Hao, Naiyuan Dong, Tingrui Leng, Yingjie Wang, Qingyu Wang, Fengxiang Yin

**Affiliations:** 1 College of Plant Science, Jilin University, Changchun, Jilin, China; 2 Baicheng Academy of Agricultural Sciences, Baicheng, Jilin, China; 3 Changchun Office of American Pioneer Thoroughbred International Companies, Changchun, Jilin, China; Duzce Universitesi, TURKEY

## Abstract

Mung bean (*Vigna radiata* L.) is an important edible bean in the human diet worldwide. However, its growth, development, and yield may be restricted or limited by insufficient or unbalanced nitrogen (N), phosphorus (P), and potassium (K) fertilization. Despite this, there are few long-term studies of the effects of varying levels of N, P, and K combined fertilizers and the optimal fertilization for improving mung bean yield and quality. This study was conducted to optimize the fertilization strategies for high yield and to improve yield components (pods per plant, seeds per pod, and 100-seed weight) in the Bailv9 mung bean cultivar, 23 treatments were tested in 2013–2015, using a three-factor (N, P, and K fertilizers), five-level quadratic orthogonal rotation combination design. Our studies showed that, the N, P, and K fertilizers significantly influenced the pods per plant and yield, which increased and then decreased with the increasing N, P, and K fertilizers. The 100-seed weight was significantly affected by the N and P fertilization, and it was increased consistently with the increasing N fertilizer, and decreased significantly with the increasing P fertilizer. Whereas, the seeds per pod significantly decreased with the increasing N and K fertilizers, and the P fertilizer had no significant effect on it. The NP interaction had a significant effect on yield and pods per plant at high N levels, while the NK interaction had a significant but opposite effect on yield at low N levels. The optimal fertilization conditions to obtain yield >2,141.69 kg ha^-1^ were 34.38–42.62 kg ha^-1^ N, 17.55–21.70 kg ha^-1^ P_2_O_5_, and 53.23–67.29 kg ha^-1^ K_2_O. Moreover, the optimal N, P, and K fertilization interval to achieve pods per plant > 23.41 and the optimal N fertilization to achieve a 100-seed weight > 6.58 g intersected with the interval for yield, but the seeds per pod did not. The fertilizer ratio for the maximum yield was N:P_2_O_5_:K_2_O = 1:0.5:1.59. Following three years experimentation, the optimal fertilization measures were validated in 2016–2017, the results indicated that yield increased by 19.6% than that obtained using conventional fertilization. The results of this study provide a theoretical basis and technical guidance for high-yield mung bean cultivation using the optimal fertilization measures.

## Introduction

Mung bean (*Vigna radiata* L.) is a cultivated legume of the family Phaseoleae. It is an annual, herbaceous, self-pollinating plant [1] that is raised as a grain, foodstuff, beverage source, vegetable, green manure, livestock feed, and medicine in China, India, Thailand, and the Philippines [1–3]. China’s total mung bean output and export rank first in the world [4]. The total annual harvest is ~1 million tons. The export volume is ~150,000–250,000 tons. Baicheng is the main mung bean producing area in China. Its total annual output is ~100,000 tons, and its export volume is ~45% of the national total [1, 5, 6]. Therefore, high mung yield and quality are of great importance to China, and those countries that import it, because of the high demand of mung bean in various use.

Nitrogen(N), phosphorus (P), and potassium (K) are essential and present in high levels in mung bean, and play important roles in its growth, development, high yield and significantly affect many mung bean traits [7, 8, 9, 10, 11]. When soil N levels are low (total N content <0.05%), the application of a small amount of N fertilizer induces rhizobia formation and promotes the growth of strong mung bean seedlings [7]. During the early growth stages before the branches develop, mung bean cannot efficiently fix atmospheric N because it has few or no rhizobia. Increasing the application of N fertilizer during the early growth period promotes vegetative growth and creates conditions favoring high yield [12]. As the plant grows, the rhizobia increases and its ability to fix atmospheric N improves; however, during the late growth period, rhizobia activity is inhibited if excess N fertilizer is applied. In this situation, flower bud differentiation and yield formation are impeded [13]. P fertilizer promotes root growth, disease resistance, drought tolerance, and enhances nutrient and water absorption in the seedlings after they have depleted their endosperm reserves [14, 15]. K fertilizer improves sugar metabolism, enhances osmotic cell concentration, maintains stomatal guard cell turgor, helps regulate stomatal opening, participates in photosynthesis, enhances drought resistance, and increases yield [16].

Appropriate use of fertilizers is of great importance to crop growth and productivity [8, 17]; however, mung bean growth and development have been seriously affected, and its yield and quality have declined, as a consequence of low fertilization levels and imbalanced N, P, and K fertilization [18]. Moreover, excessive fertilizer application has affected agricultural product quality, altered soil microecology, and enhanced soil-borne diseases [19]. Mung bean yield and quality, therefore, can be improved by the balanced use of fertilizers and by properly managing manure use [20].

Our study was conducted to determine the effects of N, P, and K fertilizers and the interactions among the three nutrients on yield and yield components. To test the changes trend and the maximum values of yield and its components with different N, P, and K levels. To generate the high yield and to improve yield components via effective and balanced fertilization. The optimal fertilization measures were established at an appropriate N, P, and K interval for yield and yield components. This study provides support for efficient cultivation of mung bean and to guide the production of mung bean.

## Materials and methods

### Experimental site

Field trials were performed in 2013–2015 at the Baicheng Academy of Agricultural Sciences, Baicheng (45.62°N; 122.81°E), Jilin Province, China. This region has the climate characteristics of plains. It has a daily mean temperature of 20°C (0.8°C above the average for the area), an annual sunshine duration of 1,243.2 h, and an annual mean rainfall of 404.9 mm. The relative soil water content was >60% during the growing periods. During the trial period of 2013, the rainfall in August was significantly lower than it was in the perennial years. In July and August of 2014 and 2015, there was less rainfall than there was in the perennial years (S1 Fig). Consequently, irrigation was performed once in August 2013 and then again in July and August of 2014 and 2015. The soil is a light chernozem with pH 7.5. The 15-mm soil layer contains 2.21% organic matter, a total N content of 0.19%, a total P content of 0.14%, a total K content of 1.93%, 120 ppm available N, 82 ppm available P, and 140 ppm available K.

### Experimental materials and design

The Bailv9 mung bean variety has a high yield, good quality, and drought tolerance. It is widely planted locally and was bred by the Baicheng Academy of Agricultural Sciences (Baicheng, Jilin Province, China). N fertilizer (urea containing 46% N), P fertilizer (calcium superphosphate containing 12% P_2_O_5_), and K fertilizer (potassium sulfate containing 50% K_2_O) were obtained from Sinochem Jilin Changshan Fertilizer Co., Ltd. (Song Yuan, Jilin Province, China).

Field experiments were conducted using N, P, and K fertilizers at five levels (Table 1). A three-factor, quadratic orthogonal rotation combination design was used for the application of the N, P, and K fertilizers in a total of 23 treatments (Table 2). All treatments were arranged in a completely randomized block with three replications for a total of 69 trial plots. Each plot was 5 m long, 2.4 m wide, and had an area of 12 m^2^. Four rows were spaced ~60 cm apart. The row spacing was 15 cm. Ten seedlings were sown per meter. The plants were thinned at the two-leaf stage to a uniform density of 160,000 plants ha^−1^. The fertilizers were mixed and sprayed as a basal fertilizer to a depth of 15 cm when the seeds were sown (Table 2).

**Table 1:** Coding design table of each N, P, and K factor level.

Levels	N (kg ha^-1^)	P_2_O_5_(kg ha^-1^)	K_2_O(kg ha^-1^)
Code mark	X_1_	X_2_	X_3_
Star on the arms (+1.68)	52.5	26.0	83.0
Upper level (+1)	41.9	20.7	66.5
Zero level (0)	26.3	13.0	41.7
Lower level (-1)	10.7	5.3	16.9
Under the arms (-1.68)	0	0	0
Change interval	15.6	7.7	24.8
Code formula	X_1j_ = (X_1_-26.3)/15.6	X_2j_ = (X_2_-13)/7.7	X_3j_ = (X-41.7)/24.8

**Table 2:** Quadratic orthogonal rotation combination design and results.

Treatments	Code gradients (Factor values kg ha^-1^)			Yield(kg ha^-1^)				Pods per plant(Pod)				Seeds per pod (Grain)				100-seed weight (g)			
	X_1_ (N)	X_2_ (P_2_O_5_)	X_3_ (K_2_O)	2013	2014	2015	Average	2013	2014	2015	Average	2013	2014	2015	Average	2013	2014	2015	Average
1	1 (41.9)	1 (20.7)	1 (66.5)	2,385.0	2,310.0	2,583.3	2,426.11 aA	28.89	27.30	30.73	28.97 aA	12.52	12.40	12.00	12.31 cdeC	6.93	6.87	6.85	6.88 bB
2	1 (41.9)	1 (20.7)	-1 (16.9)	1,991.7	2,353.3	2,208.3	2,184.44 abcdABCDEF	26.33	25.33	26.52	26.06 abcABCD	13.71	13.11	13.50	13.44 abcdeABC	6.73	6.67	6.75	6.72 cdCD
3	1 (41.9)	-1 (5.3)	1 (66.5)	2,176.7	2,076.7	2,130.0	2,127.78abcdeABCDEFG	22.78	22.78	23.72	23.09 cdefCDEF	13.11	13.22	13.80	13.38 abcdeABC	7.16	7.16	7.26	7.19 aA
4	1 (41.9)	-1 (5.3)	-1 (16.9)	1,876.7	1,970.0	1,811.7	1,886.11 cdefCDEFG	20.22	21.67	19.80	20.56 efgEFG	12.70	12.67	13.30	12.89 bcdeABC	7.29	7.15	7.06	7.17 aA
5	-1 (10.7)	1 (20.7)	1 (66.5)	2,336.7	2,216.7	1,893.3	2,148.89abcdeABCDEFG	19.67	20.67	21.50	20.61 efgEFG	12.23	12.22	12.30	12.25 eC	6.21	6.13	6.25	6.20 jkIJ
6	—1 (10.7)	1 (20.7)	-1 (16.9)	2,006.7	1,736.7	1,593.3	1,778.89 efFG	14.22	16.22	17.23	15.89 hI	13.82	13.78	14.40	14.00 abAB	6.13	6.23	6.38	6.25 hijkHIJ
7	-1 (10.7)	-1 (5.3)	1 (66.5)	2,010.0	2,046.7	2,133.3	2,063.3abcdefABCDEFG	19.67	19.67	21.78	20.37 fgFGH	12.50	12.60	12.70	12.60 cdeBC	6.28	6.35	6.21	6.28 ghijkGHIJ
8	-1 (10.7)	-1 (5.3)	-1 (16.9)	1,533.3	1,826.7	1,785.0	1,715.00 fG	16.00	16.97	16.39	16.45 hHI	13.91	14.34	14.80	14.35 aA	6.17	6.36	6.14	6.22 ijkHIJ
9	-1.68 (0)	0 (13)	0 (41.7)	1,936.7	1,686.7	1,800.0	1,807.78defEFG	20.56	18.56	21.30	20.14 fgFGH	14.76	14.22	13.96	14.31 aA	6.07	6.08	6.14	6.10 kJ
10	1.68 (52.5)	0 (13)	0 (41.7)	1,980.0	2,320.0	2,243.3	2,181.11 abcdeABCDEF	25.11	25.11	28.59	26.27 abcABCD	12.50	13.22	12.80	12.84 bcdeBC	7.38	7.26	7.18	7.27 aA
11	0 (26.3)	-1.68 (0)	0 (41.7)	1,913.3	1,863.3	1,951.7	1,909.44 bcdefBCDEFG	23.56	23.56	21.53	22.88 cdefCDEF	13.39	13.33	12.20	12.97 bcdeABC	6.78	6.88	6.66	6.77 bcBC
12	0 (26.3)	1.68 (26)	0 (41.7)	2,298.3	2,226.7	2,223.3	2,249.44 abcABCDE	23.22	25.22	24.70	24.38 bcdBCDE	12.65	12.44	12.30	12.46 cdeC	6.37	6.26	6.45	6.36 fghijFGHIJ
13	0 (26.3)	0 (13)	-1.68 (0)	1,703.3	1,955.0	1,853.3	1,837.22 defDEFG	18.78	19.33	16.36	18.16 ghGHI	13.82	13.44	12.98	13.41 abcdeABC	6.75	6.73	6.75	6.74 bcBCD
14	0 (26.3)	0 (13)	1.68 (83)	2,105.0	2,171.7	2,250.0	2,175.56abcdeABCDEFG	23.00	21.76	22.80	22.52 defDEF	12.15	12.11	12.60	12.29 deC	6.56	6.61	6.46	6.54 cdefCDEFG
15	0 (26.3)	0 (13)	0 (41.7)	2,300.0	2,251.7	2,305.0	2,285.56 abcABCD	26.44	27.32	26.23	26.66 abABC	12.92	13.89	13.20	13.34 abcdeABC	6.53	6.62	6.65	6.60 cdeCDEF
16	0 (26.3)	0 (13)	0 (41.7)	2,345.0	2,346.7	2,156.7	2,282.78 abcABC	25.22	27.22	25.80	26.08 abcABCD	13.82	13.89	13.00	13.57 abcABC	6.43	6.49	6.51	6.48 efghDEFGH
17	0 (26.3)	0 (13)	0 (41.7)	2,361.7	2,320.0	2,250.0	2,310.56 abABC	23.78	24.22	23.82	23.94bcdeBCDEF	13.12	13.89	13.70	13.57 abcABC	6.43	6.45	6.50	6.46 efghEFGHI
18	0 (26.3)	0 (13)	0 (41.7)	2,365.0	2,458.3	2,296.7	2,373.33 aAB	24.11	23.11	26.72	24.65 bcdBCD	13.65	13.56	11.80	13.00 bcdeABC	6.51	6.56	6.55	6.54 cdefCDEFG
19	0 (26.3)	0 (13)	0 (41.7)	2,378.3	2,375.0	2,245.0	2,332.78 aABC	26.89	25.89	24.54	25.77 abcdABCD	13.21	12.91	13.30	13.14 abcdeABC	6.46	6.44	6.55	6.48 defgDEFGH
20	0 (26.3)	0 (13)	0 (41.7)	2,255.0	2,448.3	2,176.7	2,293.33 abABCD	25.56	25.56	26.83	25.98 abcdABCD	13.72	13.67	13.20	13.53 abbcdABC	6.51	6.49	6.62	6.54 cdefCDEFG
21	0 (26.3)	0 (13)	0 (41.7)	2,226.7	2,243.3	2,485.0	2,318.33 aABC	26.11	27.56	28.23	27.30 abAB	13.81	13.56	13.30	13.56 abcdABC	6.42	6.54	6.40	6.45 efghiEFGHI
22	0 (26.3)	0 (13)	0 (41.7)	2,228.3	2,253.3	2,388.3	2,290.00 abcABCD	25.33	26.22	25.12	25.56 abcdABCD	13.81	13.67	13.20	13.56 abcdABC	6.54	6.55	6.56	6.55 cdefCDEF
23	0 (26.3)	0 (13)	0 (41.7)	2,365.0	2,083.3	2,395.0	2,281.11 abcABCD	26.22	26.22	26.19	26.21 abcABCD	12.92	12.89	12.50	12.77 bcdeBC	6.39	6.49	6.50	6.46 efghEFGH

Note: N: urea (N = 46%), P: calcium superphosphate (P_2_O_5_ = 12%), K: potassium sulfate (K_2_O = 50%). Variance analysis results showed annual *P* values of 0.8524, 0.1523, 0.2475, and 0.7331. Differences were not significant. For the treatments with *P* = 0.0001, the differences were significant.

Differences in the mean values of each treatment with a, b, c… were significant (*P*<0.05).

Differences in the mean values of each treatment with A, B, C… were extremely significant (*P* <0.01).

### Measurement of mung bean yield and yield components

All plants within a 6m^2^ area (two 5-m long rows) of each plot were hand-harvested at maturity. The seeds were first dried to <13% moisture before yield determination. A 6m^2^ sample area was used to measure the yield per hectare. The pods per plant, seeds per pod, and the 100-seed weight were measured on five plants per plot. The pods per plant was calculated from the average pods of five samples. The seeds per pod was calculated by randomly counting the seeds in ten mature pods using the average number of grains. For 100-seed weight, 100 seeds were weighed three times and the average weight was calculated. The error was not allowed to exceed 0.5 g.

### Implementation and validation of optimized fertilization measures

The comparison of optimal and conventional fertilization was carried out in the main mung bean production areas of Baicheng in Jilin Province and Zhenlai and Taikang in Heilongjiang Province during 2016–2017. Baicheng had more favorable water and fertilizer conditions and a light chernozem soil whereas Zhenlai and Taikang had relatively poorer water and fertilizer conditions and sandy loam. The optimized fertilizers contained 74.8 kg ha^-1^ of urea, 44 kg ha^-1^ of diammonium phosphate, and 133 kg ha^-1^ of potassium sulfate (N:P_2_O_5_:K_2_O = 1:0.5:1.6). The conventional fertilizers contained 200 kg ha^-1^ of compound fertilizer (N:P_2_O_5_:K_2_O = 1:1:1). Each plot was 0.5 ha and the Bailv9 mung bean was used as the experimental material.

### Statistical analysis

Differences in the values for the three trial years were not significant (*P*>0.05); however, the relative effects of the various N (X_1_), P (X_2_), and K (X_3_) treatments were significantly different (*P*<0.01) (Table 2). A regression analysis was therefore performed using the means for the three trial years. The regression equation was established for the corresponding tests of the effects of N, P, and K fertilizers on yield and yield components. Data Processing System (DPS) software (Hangzhou Ruifeng Information Technology Co., Ltd., Hangzhou, China) was used for mathematical and statistical analysis.

## Results

### Effects of N, P, and K fertilizers on yield

The regression equation for the correlation between N, P, K fertilizers and expected yield was as follows: Y = 2306.85 + 113.22X_1_ + 96.50X_2_ + 129.66X_3_−104.14X_1_^2^–74.09X_2_^2^–99.92X_3_^2^ + 55.90X_1_X_2_−29.37X_1_X_3_ + 2.71X_2_X_3_. The *P* value for the regression, which was extremely significant (*P*<0.01), indicating good model fitness. The *P* value for the lack of fit and was, therefore, not significant (*P*>0.05) (Table 3). This finding suggests that unknown factors marginally affected the experimental results. The regression model, therefore, was relatively suitable for evaluating the effects of N, P, and K fertilizers on yield.

**Table 3:** Analysis of variance of the effects of N, P, and K fertilizers on yield and yield components.

Source of variation	Degrees of freedom	Yield			Pods per plant			Seeds per pod			100- seeds weight		
		Mean square	F-value	*P*-value	Mean square	F-value	*P*-value	Mean square	F-value	*P*-value	Mean square	F-value	*P*-value
X_1_	1	175,056.1	119.4439	0.0001**	93.1624	62.8561	0.0001**	0.9767	8.5414	0.0119*	1.8143	403.5718	0.0001**
X_2_	1	127,182.4	86.7789	0.0001**	13.5089	9.1144	0.0099**	0.3161	2.7643	0.1203	0.1647	36.6250	0.0001**
X_3_	1	229,579.6	156.6464	0.0001**	33.5729	22.6514	0.0004**	2.6568	23.234	0.0003**	0.0016	0.3489	0.5649
X_1_^2^	1	172328.8	117.5831	0.0001**	17.7724	11.9910	0.0042**	0.1904	1.6654	0.2194	0.0537	11.9430	0.0043**
X_2_^2^	1	87225.8	59.5158	0.0001**	13.0811	8.8257	0.0108*	0.6016	5.2609	0.0391*	0.0039	0.8716	0.3675
X_3_^2^	1	158635.5	108.2399	0.0001**	68.1191	45.9595	0.0001**	0.3426	2.9965	0.1071	0.0283	6.3001	0.0261*
X_1_X_2_	1	24,999.6	17.0577	0.0012**	17.1112	11.5448	0.0048**	0.0041	0.0354	0.8536	0.0630	14.016	0.0025**
X_1_X_3_	1	6,902.5	4.7097	0.0491*	1.28	0.8636	0.3697	1.0225	8.9414	0.0104*	0.0036	0.8036	0.3863
X_2_X_3_	1	58.7	0.0401	0.8445	0.174	0.1174	0.7373	0.3281	2.8688	0.1141	0.0001	0.0250	0.8767
Regression	9	108,493.1	F_2_ = 74.02	0.0001**	28.5216	F_2_ = 19.2433	0.0001**	0.7157	F_2_ = 6.2584	0.0047**	0.2369	F_2_ = 52.701	0.0001**
Residual	13	1,465.6			1.4822			0.1144			0.0045		
Lack of fit	5	2,331.5	F_1_ = 2.522	0.0831	2.2318	F_1_ = 2.20189	0.1168	0.1535	F_1_ = 1.7073	0.2022	0.0073	F_1_ = 2.6893	0.07
Error	8	924.4			1.0136			0.0899			0.0027		
Total	22												

Note: X_1_, X_2_, and X_3_ represent the N, P, and K fertilizers, respectively.

* were significant (*P*<0.05).

** were extremely significant (*P*<0.01).

The absolute values of the regression coefficients indicated that the relative influences of the N, P, and K fertilizers on yield were as follows: K > N > P. The relative magnitudes of the interaction effects of the three nutrients were NP > NK > PK. N, P, and K fertilizers all had extremely significant *(P*<0.01) effects on yield, while the interactions between the N and P, N and K and P and K fertilizers had extremely significant (*P*<0.01) effect, significant effect (*P*<0.05) and no significant effect (*P*>0.05) on yield, respectively (Table 3).

**Fig 1:**
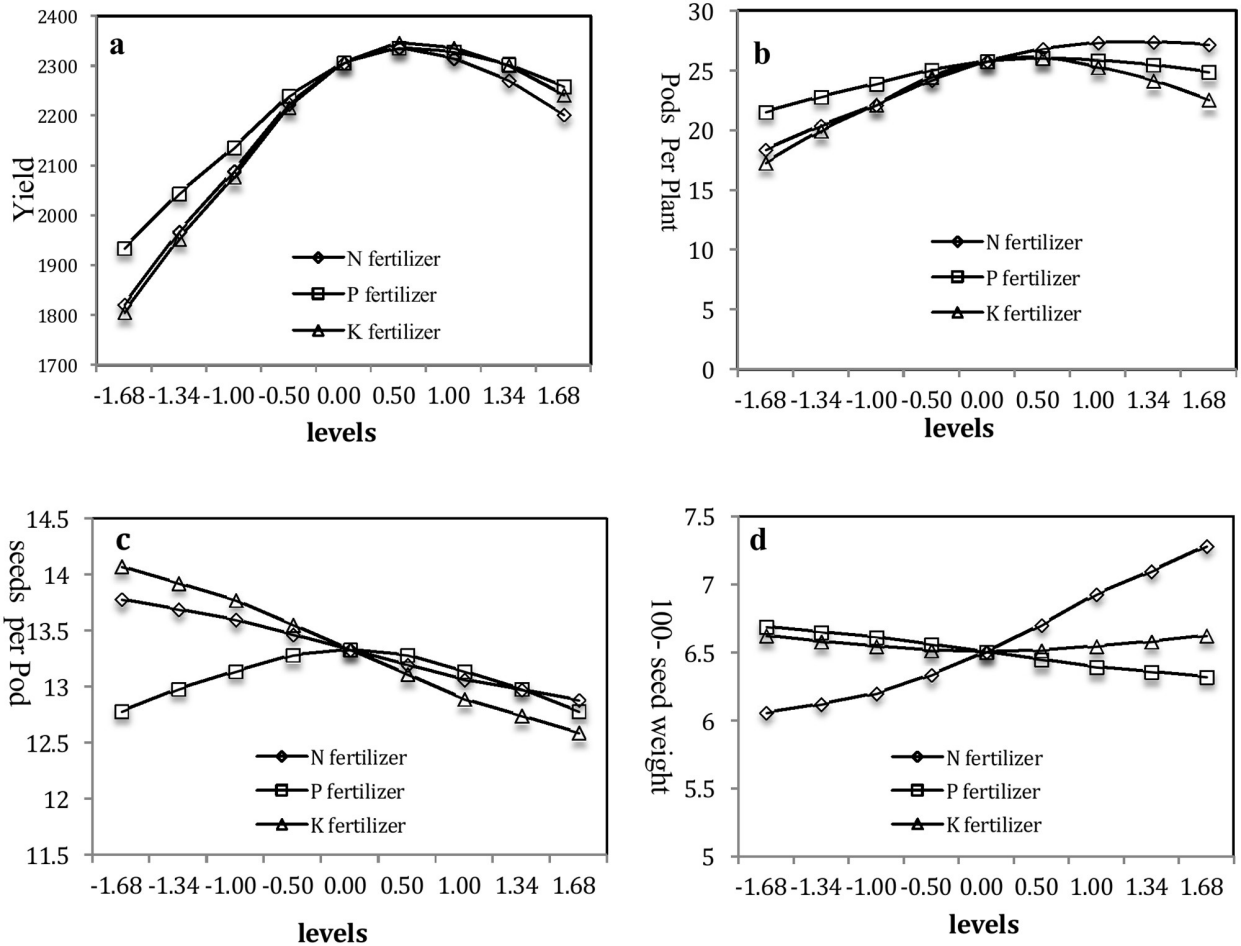
Effect of N, P, and K fertilizers on yield and yield components. a. Effects of N, P, and K fertilizers on yield when N(X_1_) was within (-1.68, 1.68), Y = 2,306.85 + 113.22X_1_−104.14X_1_^2^. When P(X_2_) was within (-1.68, 1.68), Y = 2306.85 + 96.50X_2_−74.09X_2_^2^. When K(X_3_) was within (-1.68, 1.68), Y = 2306.85 + 129.66X_3_−99.92X_3_^2^. b. Effects of N, P, and K fertilizers on pods per plant. When N(X_1_) was within (-1.68, 1.68), Y = 25.81 + 2.61X_1_−1.06X_1_^2^. When P(X_2_) was within (-1.68, 1.68), Y = 25.81 + 0.99X_2_−0.91X_2_^2^. When K(X_3_) was within (-1.68, 1.68), Y = 25.81 + 1.57X_3_−2.07X_3_^2^. c. Effects of single-factor N, P, and K fertilizer on the seeds per pod. When N(X_1_) was within (-1.68, 1.68), Y = 13.34–0.27X_1_ + 0.11X_1_^2^. When P(X_2_) was within (-1.68, 1.68), Y = 13.34–0.15X_2_−0.19X_2_^2^. When K(X_3_) was within (-1.68, 1.68), Y = 13.34–0.44X_3_−0.15X_3_^2^. d. Effects of N, P, and K fertilizers on 100-seed weight. When N(X_1_) was within (-1.68, 1.68), Y = 6.51 + 0.36X_1_ + 0.06X_1_^2^. When P(X_2_) was within (-1.68, 1.68), Y = 6.51–0.11X_2_ + 0.02X_2_^2^. When K(X_3_) was within (-1.68, 1.68), Y = 6.51–0.01X_3_ + 0.04X_3_^2^.

As shown in Fig 1a, when the N, P, and K fertilizers were in the range of -1.68–0.5, the yield sharply increased with N, P, and K fertilizers and then slowly decreased at levels within the range of 0.5–1.68. The maximum value was 2,337.42 kg ha^-1^ at the 0.5 level of 34.10 kg ha^-1^ N, 2,336.58 kg ha^-1^ at the 0.5 level of 16.85 kg ha^-1^ P_2_O_5_, and 2,346.70 kg ha^-1^ at the 0.5 level of 54.10 kg ha^-1^ K_2_O.

**Fig 2:**
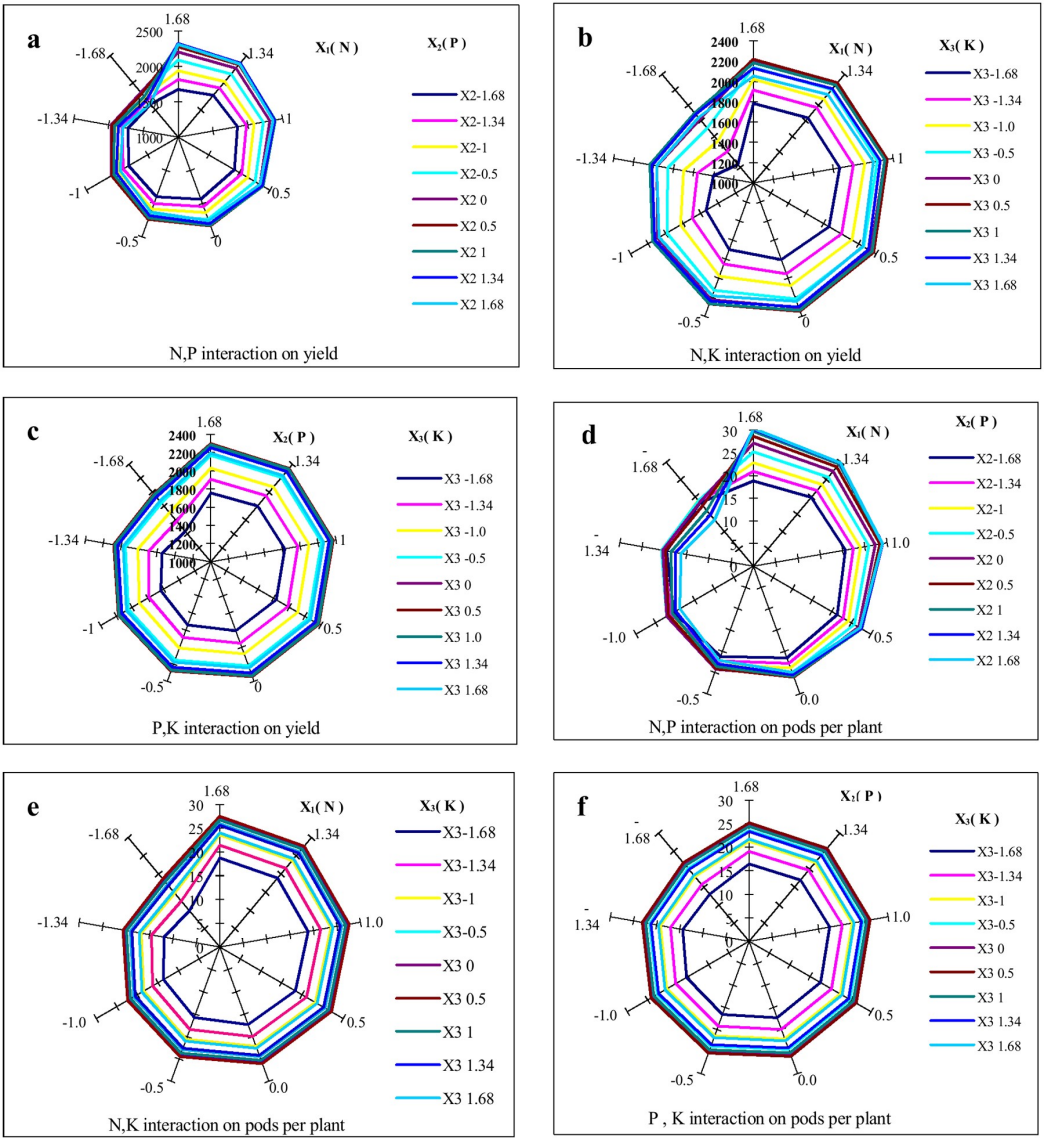
Effects of N(X_1_), P(X_2_), and K(X_3_) interactions on pods per plant and yield. The radiation line represents the yield value (Y kg ha^-1^) and pods per plant. a. Effects of N and P fertilizer interaction on yield (X_3_ = 0). The radiation angle indicates N fertilizer levels and the series indicates the P fertilizer levels. b. Effects of N and K fertilizer interaction on yield (X_2_ = 0). The radiation angle indicates the N fertilizer levels and the series indicates the K fertilizer levels. c. Effects of P and K fertilizer interaction on yield (X_1_ = 0). The radiation angle indicates the P fertilizer levels and the series indicates the K fertilizer levels. d. Effects of N and P fertilizer interaction on pods per plant. The radiation angle indicates the N fertilizer levels and the series indicates the P fertilizer levels (X_3_ = 0). e. Effects of N and K fertilizer interaction on pods per plant. The radiation angle indicates the N fertilizer levels and the series indicates the K fertilizer levels (X_2_ = 0). f. Effects of P and K fertilizer interaction on pods per plant (X_1_ = 0). The radiation angle indicates the P fertilizer levels and the series indicates K fertilizer levels.

As shown in Fig 2a, the yield slowly increased at low values as N and P fertilizers increased when the N fertilizer levels were <0 (26.3 kg ha^-1^ N), and the yield differences were not significant. The interaction between N and P fertilizers, therefore, had no significant effect on the yield when the N fertilizer levels were <0; however, yield significantly increased at high values with increasing N and P fertilizers when the N fertilizer levels were >0, and yield differences were significant. Therefore, the interaction between N and P fertilizers had an extremely significant (*P*<0.01) effect on yield at N fertilizer levels >0. The maximum yield was 2,394.24 kg ha^-1^ at the 1.0 level of 41.9 kg ha^-1^ N and at the 1.0 level of 20.7 kg ha^-1^ P_2_O_5_ (Fig 2a). As shown in Fig 2b, the N and K interaction effect indicated that yield significantly increased at high values as N and K fertilizers increased when the N fertilizer levels were <0 (26.3 kg ha^-1^ N). Yield differences were extremely significant. Therefore, the interaction between the N and K fertilizers had a significant (*P*<0.05) effect on yield when the N fertilizer levels were <0; yield, however, increased non-significantly at a high value with increasing N and K fertilizers when the N fertilizer levels were >0. Yield differences were not significant. Therefore, the interaction between N and K fertilizers had no significant (*P*>0.05) effect on yield at N fertilizer levels >0. The maximum yield was 2369.93 kg ha^-1^ at the 0.5 level of 34.10 kg ha^-1^ N and 54.10 kg ha^-1^ K_2_O (Fig 2b). The PK interaction, however, had no significant (*P*>0.05) effect on yield. (Fig 2c).

### Effects of N, P, and K fertilizers on pods per plant

The regression equation for the relationship between the N, P, K fertilizers and pods per plant was as follows: Y = 25.81 + 2.61X_1_ + 0.99X_2_ + 1.57X3−1.06X_1_2–0.91X_2_2–2.07X_3_^2^ + 1.46X_1_X2−0.40X_1_X_3_ + 0.15X_2_X_3_. The *P* valule for the regression, which was extremely significant,(*P*<0.01), so the regression model was a good fit for the experiment. The *P* value for the lack of fit and so was not significant (*P*>0.05). The external factors, therefore, had negligible influences on the experimental results (Table 3). The regression model was suitable for evaluating the effects of N, P, and K fertilizers on pods per plant.

According to the absolute value of the regression coefficient, the relative influence of N, P, and K fertilizers on pods per plant was as follows: N > K > P. The relative influence of the interaction effects of the three nutrients on pods per plant was NP > NK > PK. N, P, and K fertilizers all had extremely significant (*P*<0.01) effects on pods per plant. The interaction between N and P fertilizer had an extremely significant (*P*<0.01) effect on pods per plant, but the interactions between N and K and between P and K fertilizers had no significant (*P*>0.05) effects on pods per plant (Table 3).

As shown in Fig 1b, pods per plant sharply increased with N fertilizer at levels <0.5 (34.10 kg ha^-1^ N) and then slowly increased with N fertilizer at levels >0.5, and presented a tiny decrease at the point of 1.68 level. Pods per plant gradually increased with P fertilizer at levels <0.5 and gradually decreased with increasing P fertilizer at levels >0.5. Moreover, pods per plant sharply increased with K fertilizer at levels <0.5, but when K fertilizer levels were >0.5, pods per plant slowly decreased with increasing K fertilizer. The maximum values were 27.41 at the 1.34 level of 47.20 kg ha^-1^ N, 26.08 at the 0.5 level of 16.85 kg ha^-1^ P_2_O_5_, and 26.08 at the 0.5 level of 54.10 kg ha^-1^ K_2_O.

As shown in Fig 2d, pods per plant slowly increased at low value as N and P fertilizer increased when the N fertilizer levels were <0 (26.3 kg ha^-1^ N) and the differences in the pods per plant were not significant. Therefore, the interaction between the N and P fertilizer had no significant (*P*>0.05) effect on pods per plant at N fertilizer levels <0; however, when N fertilizer levels were >0, pods per plant significantly increased at high values with increasing N and P fertilizer and the differences in the pods per plant was significant. The aforementioned results, therefore, suggested that the interaction between the N and P fertilizers had extremely significant (*P*<0.01) effect on pods per plant at N fertilizer levels >0. The maximum pods per plant was 30.45 at the 1.68 levels of 52.5 kg ha^-1^ N and 26 kg ha^-1^ P_2_O_5_ (Fig 2d). The interaction between the N and K and P and K fertilizers, however, had no significant (*P*>0.05) effect on pods per plant (Fig 2e and 2f).

### Effects of N, P, and K fertilizers on seeds per pod

The regression equation for the correlation between the N, P, K fertilizers and the seeds per pod was as follows: Y = 13.33–0.27X1−0.15X2−0.44X_3_ + 0.11X_1_2–0.19X_2_2–0.15X_3_^2^ + 0.02X_1_X_2_ + 0.36X_1_X3−0.20X_2_X_3_. The *P* value for the regression, which was extremely significant (*P*<0.01); therefore, the regression model was a good fit for the experimental results. The *P* value for the lack of fit, which was not significant (*P*>0.05); therefore, unknown factors slightly influenced the regression model (Table 3). The regression model could be used to evaluate the effects of N, P, and K fertilizers on seeds per pod.

According to the absolute value of the regression coefficient, the relative effects of the N, P, and K fertilizers on seeds per pod were in the order K > N > P. The relative interaction effects among the three fertilizers were in the order NK > PK > NP. N and K fertilizers had extremely significant (*P*<0.01) effects on seeds per pod, but the P fertilizer had no significant (*P*>0.05) effect. The interaction between N and K fertilizer significantly (*P*<0.05) affected the seeds per pod. The interactions between N and P and between P and K fertilizers had no significant (*P*>0.05) effects on the seeds per pod (Table 3).

As shown in Fig 1c, the seeds per pod sharply decreased with increasing N and K fertilizer levels; however, as the levels of P fertilizer increased, the seeds per pod sharply increased then gradually decreased. The maximum seeds per pod were 13.79 at the -1.68 level of 0 kg ha^-1^ N, 13.29 at the 0 level of 13 kg ha^-1^ P_2_O_5_, and 14.07 at the -1.68 level of 0 kg ha^-1^ K_2_O_5_.

**Fig 3:**
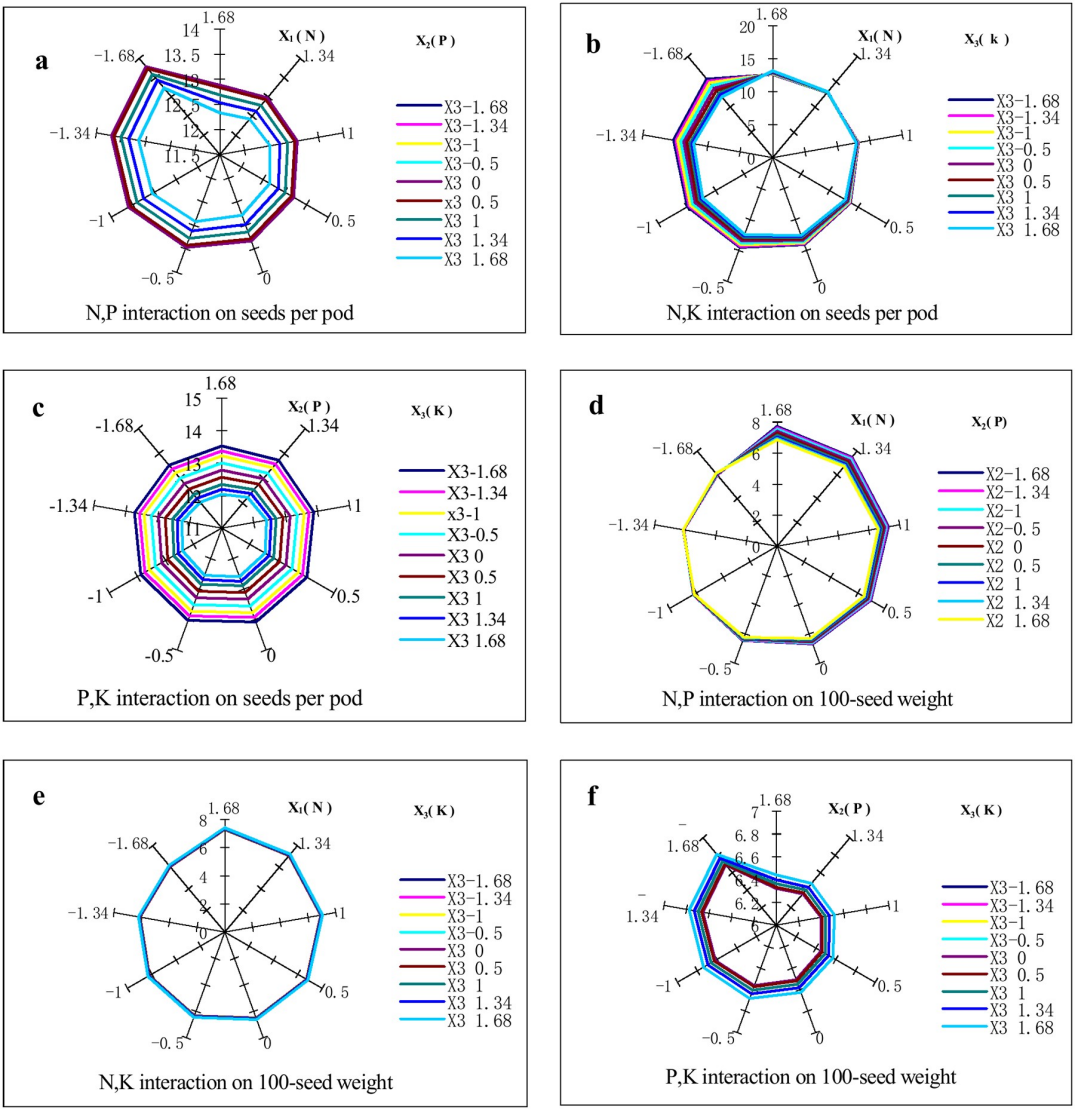
Effects of N(X_1_), P(X_2_), and K(X_3_) interactions on 100-seed weight and seeds per pod. The radiation line represents the seeds per pod and 100-seed weight (g). a. Effects of N and P fertilizer interaction on the seeds per pod (X_3_ = 0). The radiation angle indicates the N fertilizer levels and the series indicates the P fertilizer levels. b. Effects of N and K fertilizer interaction on the seeds per pod (X_2_ = 0). The radiation angle indicates the N fertilizer levels and the series indicates the K fertilizer levels. c. Effects of P and K fertilizer interaction on the seeds per pod (X_1_ = 0). The radiation angle indicates the P fertilizer levels and the series indicates the K fertilizer levels. d. Effects of N and P fertilizer interaction on 100-seed weight. The radiation angle indicates the N fertilizer levels and the series indicates the P fertilizer levels (X_3_ = 0). e. Effects of N and K fertilizer interaction on 100-seed weight. The radiation angle indicates the N fertilizer levels and the series indicates the K fertilizer levels (X_2_ = 0). f. Effects of P and K fertilizer interaction on 100-seed weight (X_1_ = 0). The radiation angle indicates the P fertilizer levels and the series indicates the K fertilizer levels.

As shown in Fig 3b, the seeds per pod significantly differed with increasing N and K fertilizer levels when the N fertilizer levels were <0.5 but did not significantly differ at N levels >0.5. Therefore, the interaction between N and K fertilizers significantly (*P*<0.05) affected the seeds per pod at N fertilizer levels <0.5. The maximum value was 15.54 at the -1.68 level of both 0 kg ha^-1^ N and K_2_O; The interactions between the N and P and P and K fertilizers had no significant effects on the seeds per pod (Fig 3a and 3c).

### Effects of N, P, and K fertilizers on 100-seed weight

The regression equation for the correlation between N, P, K fertilizers and 100-seed weight was as follows: Y = 6.51 + 0.36X1−0.11X2−0.01X_3_ + 0.06X_1_^2^ + 0.02X_2_^2^ + 0.04X_3_2–0.09X_1_X_2_ + 0.02X_1_X_3_ + 0.003X_2_X_3_. The *P* value for the regression, which was extremely significant (*P*<0.01); therefore, the regression model fit the experimental results. The *P* value for the lack of fit, which was not significant (*P*>0.05); therefore, unknown factors had a slight effect on 100-seed weight. The regression model was a good fit for evaluating the effects of N, P, and K fertilizers on 100-seed weight (Table 3).

According to the absolute value of the regression coefficient, the relative magnitudes of the effects of N, P, and K fertilizers on the 100-seed weight were N > P > K. The relative magnitudes of the effects of the interactions among the three fertilizers were in the order NP > NK > PK. N and P fertilizers had extremely significant (*P*<0.01) effects on 100-seed weight but the K fertilizer had no significant (*P*>0.05) effect on it. The interaction between N and P fertilizer had an extremely significant (*P*<0.01) effect on 100-seed weight whereas the interactions between N and K and P and K fertilizers did not (*P*>0.05) (Table 3).

As shown in Fig 1d, the 100-seed weight slightly and then rapidly increased with increasing N fertilizer and the maximum 100-seed weight was 7.29 g at the 1.68 level of 52.5 kg ha^-1^ N. As P increased, the 100-seed weight slightly decreased but did not significantly change with increasing K. The interaction between N and P significantly affected the 100-seed weight at N >0, and the maximum value was 7.70 g at the 1.68 level of 52.5 kg ha^-1^ N and the -1.68 level of 0 kg ha^-1^ P_2_O_5_ (Fig 3d). The interactions between N and K and P and K, however, had no significant effects on the 100-seed weight (Fig 3e and 3f).

### Optimal fertilization measures for high yield and suitable yield components

#### Optimal fertilization measures for high yield

As shown in Table 4, the frequency analysis for the optimal fertilization measures showed that 30 combinations of N, P, and K fertilizers resulted in yields >2,141.69 kg ha^-1^. The 95% confidence interval of N, P_2_O_5_, and K_2_O were distributed in 0.518–1.046, 0.591–1.130, and 0.465–1.032 respectively. These data were inserted into the factor-coding formula. The optimal fertilization measures for high yield (>2,141.69 kg ha^-1^) were 34.38–42.62 kg ha^-1^ N, 17.55–21.70 kg ha^-1^ P_2_O_5_, and 53.23–67.29 kg ha^-1^ K_2_O. According to the regression analysis, the maximum yield was 2,394.6 kg ha^-1^ which corresponded to frequencies of 0.3667 (N), 0.3333 (P), and 0.3667 (K), respectively. The corresponding amounts of fertilizers were at 1.0 level of 41.9 kg ha^-1^ N, 1.0 level of 20.7 kg ha^-1^ P_2_O_5_, and 1.0 level of 66.5 kg ha^-1^ K_2_O. The best fertilizer ratio was N:P_2_O_5_:K_2_O = 1:0.5:1.59.

**Table 4:** Frequency distribution and fertilization measures for yield and yield components.

Y yield and yield components	Levels	N		P_2_O_5_		K_2_O	
		X_1_ Times	Frequency	X_2_ Times	Frequency	X_3_ Times	Frequency
Ymax = 2,394.6 kg ha^-1^		X_1_ = 1	0.3667	X_2_ = 1	0.3330	X_3_ = 1	0.3670
Yield ≥2,141.69 kg ha^-1^	-1.68	0	0	0	0	0	0
	-1	1	0.0333	1	0.0333	2	0.0667
	0	10	0.3333	9	0.3000	9	0.3000
	1	11	0.3667	10	0.3333	11	0.3667
	1.68	8	0.2663	10	0.3333	8	0.2667
	Weight mean	0.782		0.861		0.748	
	Standard error	0.135		0.138		0.145	
	95% confidence interval	0.518–1.046		0.591–1.130		0.465–1.032	
	Fertilization (kg ha^-1^)	34.38–42.62		17.55–21.70		53.23–67.29	
Ymax = 30.45		X_1_ = 1.68	0.3636	X_2_ = 1.68	0.3030	X_3_ = 0	0.3333
Pods per plant ≥23.41	-1.68	0	0	0	0	0	0
	-1	0	0	3	0.0909	6	0.1818
	0	7	0.2121	10	0.3030	11	0.3333
	1	14	0.4242	10	0.3030	10	0.3030
	1.68	12	0.3636	10	0.3030	6	0.1818
	Weight mean	1.036		0.722		0.427	
	Standard error	0.107		0.149		0.157	
	95% confidence interval	0.826–1.246		0.430–1.013		0.119–0.735	
	Fertilization (kg ha^-1^)	39.19–45.74		16.31–20.80		44.65–59.93	
Ymax = 15.54		X_1_ = -1.68	0.3750	X_2_ = 0	0.2500	X_3_ = -1.68	0.4000
Seeds per pod ≥13.2	-1.68	15	0.375	7	0.175	16	0.4
	-1	13	0.325	8	0.2	15	0.375
	0	11	0.275	10	0.25	9	0.225
	1	1	0.025	8	0.2	0	0
	1.68	0	0	7	0.175	0	0
	Weight mean	-0.931		0		-1.048	
	Standard error	0.117		0.186		0.101	
	95% confidence interval	-1.159–-0.702		-0.365-0.365		-1.246–-0.850	
	Fertilization (kg ha^-1^)	8.22–15.35		10.19–15.81		10.80–20.62	
Ymax = 7.84		X_1_ = 1.68	0.4032	X_2_ = -1.68	0.2419	X_3_ = -1.68	0.2097
100-seed weight ≥6.58 g	-1.68	0	0	15	0.2419	13	0.2097
	-1	0	0	15	0.2419	12	0.1935
	0	12	0.1935	12	0.1935	12	0.1935
	1	25	0.4032	10	0.1613	12	0.1935
	1.68	25	0.4032	10	0.1613	13	0.2907
	Weight mean	1.081		-0.216		0	
	Standard error	0.078		0.155		0.159	
	95% confidence interval	0.929–1.234		-0.521–0.088		-0.312–0.312	
	Fertilization (kg ha^-1^)	40.79–45.55		8.99–13.68		33.96–49.44	

#### Optimal fertilization measures for pods per plant

As shown in Table 4, the frequency analysis for the optimal fertilization measures showed that 33 combinations of N, P, and K fertilizers resulted in pods per plant >23.41. The 95% confidence interval of the N, P_2_O_5_, K_2_O were distributed in 0.826–1.246, 0.430–1.013, and 0.119–0.735 respectively. Therefore, the optimal fertilization measures to achieve high pods per plant (> 23.41) were 39.19–45.74 kg ha^-1^ N, 16.31–20.80 kg ha^-1^ P_2_O_5_, and 44.65–59.93 kg ha^-1^ K_2_O (Table 4). According to the regression analysis, the maximum pods per plant was 30.45 which corresponded to frequencies of 0.3636 (N), 0.3030 (P), and 0.3333 (K), respectively. The corresponding amounts of fertilizers were at the 1.68 level of 52.5 kg ha^-1^ N, the 1.68 level of 26 kg ha^-1^ P_2_O_5_, and the 0 level of 41.7 kg ha^-1^ K_2_O. The best fertilizer ratio was N:P_2_O_5_:K_2_O = 1:0.5:0.8.

#### Optimal fertilization measures for seeds per pod

As shown in Table 4, the frequency analysis of the optimal fertilization measures showed that 40 combinations of N, P, and K fertilizers resulted in seeds per pod >13.20. The 95% confidence interval of the N, P_2_O_5_, and K_2_O were distributed in -1.159 to -0.702, -0.365–0.365, and -1.246 to -0.850 respectively. The optimal fertilization measures for high seeds per pod (> 13.2) were 8.22–15.35 kg ha^-1^ N, 10.19–15.81 kg ha^-1^ P_2_O_5_, and 10.80–20.62 kg ha^-1^ K_2_O (Table 4). According to the regression analysis, the maximum seeds per pod was 15.5 which corresponded to frequencies of 0.3750 (N), 0.2500 (P), and 0.4000 (K), respectively. The corresponding amounts of fertilizers were at the -1.68 level of 0 kg ha^-1^ N, the 0 level of 13 kg ha^-1^ P_2_O_5_, and the -1.68 level of 0 kg ha^-1^ K_2_O.

#### Optimal fertilization measures for 100-seed weight

As shown in Table 4, the frequency analysis of the optimal fertilization measures showed that 62 of the N, P, and K fertilizers combinations resulted in a 100-seed weight >6.58 g. The 95% confidence interval of the N, P_2_O_5_, and K_2_O were distributed in 0.929–1.234, -0.521–0.088, and -0.312–0.312 respectively. The optimal fertilization measures for high 100-seed weight (> 6.58 g) were 40.79–45.55 kg ha^-1^ N, 8.99–13.68 kg ha^-1^ P_2_O_5_, and 33.96–49.44 kg ha^-1^ K_2_O (Table 4). According to the regression analysis, the maximum 100-seed weight was 7.84 g, which corresponded to frequencies of 0.4032 (N), 0.2419 (P), and 0.2097 (K), respectively. The corresponding amounts of fertilizers were at the 1.68 level of 52.5 kg ha^-1^ N and the -1.68 levels of both 0 kg ha^-1^ P_2_O_5_ and K_2_O.

#### Implementation and validation of the optimized fertilization

The results showed that the average yield of each site at which the optimized fertilization program was implemented reached 1,995.8 kg ha^-1^ and 1,895.8 kg ha^-1^ in 2016 and 2017 respectively, and was 18.6% and 20.6% higher in 2016 and 2017 respectively, than that obtained with conventional fertilization. Two-year multi-point tests determined an optimized fertilization program with an average yield of 1,945.8 kg ha^-1^ which was 19.6% higher than that obtained with conventional fertilization (Table 5).

**Table 5:** Implementation and verification of optimized fertilization.

Years	Location	Optimal fertilization (kg ha^1^)	conventional fertilization kg ha^1^	Percentage increase (%)
**2016**	Baicheng, Jilin Province	2,513.2	2,151.8	16.8
	Zhenlai, Jilin Province	1,795.8	1,518.9	18.2
	Taikang, Heilongjiang Province	1,678.5	1,376.7	21.9
**Average**		1,995.8	1,682.5	18.6
**2017**	Baicheng, Jilin Province	2,392.7	2,018.6	18.5
	Zhenlai, Jilin Province	1,713.2	1,386.9	23.5
	Taikang, Heilongjiang Province	1,581.5	1,309.7	20.8
**Average**		1,895.8	1,571.7	20.6
**Two-year average**		1,945.8	1,627.1	19.6

## Discussion

The main objective of the mung bean research was to optimize yield and quality [21, 22, 23]. Greater pods per plant, seeds per pod, and higher stable grain weight are all indices of high grain quality [24]. The pods per plant, seeds per pod, and 100-seed weight are essential yield components [25]. Therefore, we investigated yield and yield components in this study. Previous reports, however, have showed that yield, pods per plant, seeds per pod, and 100-seed weight of mung bean are significantly affected by the application of N, P, and K fertilizers [26]. These results were demonstrated in our studies, and we found the different effects of N, P, and K fertilizers and their interactions.

Previous studies suggested that N fertilization was the most important factor in mung bean production [27]. In the present study, the N fertilizer significantly influenced the yield, pods per plant, seeds per pod, and 100-seed weight. Previous reports showed that, increasing the amount of N fertilizer at early growth stages promotes vegetative growth and creates conditions conducive to high yield. As the plants grew, however, rhizobia gradually improves their ability to fix atmospheric N [12] and yield decreases with increasing N application rate [28]. Our results corroborated the findings of these studies, as they demonstrated that the yield rapidly increased and then gradually decreased with increasing N fertilization (Fig 1a). The 100-seed weight was significantly influenced by N fertilization in common bean (*Phaseolus vulgaris*) [29]. In this experiment, it was found that, the 100-seed weight consistently increased with increasing N fertilizer. Therefore, N fertilizer significantly influenced grain weight, size, and fullness as well as yield. Previous studies have showed that N fertilization does not significantly affect the seeds per pod but dose significantly influenced the pods per plant [29]. In our study, N fertilization significantly enhanced the pods per plant, which rapidly increased and then gradually decreased with increasing N fertilizer rate; however, seeds per pod decreased with increasing N fertilizer rate. Therefore, the appropriate N fertilizer application rate should be determined for yield, pods per plant, and 100-seed weight.

According to our results, P fertilizer had significant effects on yield, pods per plant, and 100-seed weight. P deficiency suppresses growth and lowers yield, whereas, excessive amounts of P delays maturation and seed set [30]. This finding was also demonstrated in our study, the yield and pods per plant increased and then gradually decreased with increasing P fertilizer. In contrast, the 100-seed weight slightly decreased with increasing P fertilizer; however, the P fertilizer effect on seeds per pod was non-significant. Therefore, the appropriate P fertilizer application rate should be determined for yield, pods per plant.

K fertilizer significantly influenced yield, pods per plant, and seeds per pod in dry bean (*P*. *vulgaris*) [31]. In our study, the yield and pods per plant rapidly increased and then gradually decreased with increasing K fertilizer. The seeds per pod decreased with increasing K fertilizer; however, the 100-seed weight did not significantly change with K fertilizer rate. Therefore, the appropriate K fertilizer application rate should be determined for yield and pods per plant.

In this study, the effects of the NK interaction on yield was opposite to those of the NP interaction effect (Fig 2a and 2b). The effects of the NK interaction, however, were significant at low N fertilizer levels; those of the NP interaction were significant at high N fertilizer levels. Moreover, the NP interaction had the same presentation of effect on the pods per plant and the yield. These findings suggested that, the NP and NK interactions were effective at the goal of achieving high yield, and the NP interaction was effective at achieving a high pods per plant. The effects of interactions of N, P, and K fertilizers on the 100-seed weight and the seeds per pod should investigate progressively in the future.

The results reported herein suggested that the optimal fertilization for high yield should be identified at the appropriate intervals, but was not only determined by integrating the fertilization optima for each yield component. Our results showed that the optimal fertilization measures for yield > 2,141.69 kg ha^-1^ were 34.38–42.62 kg ha^-1^ N, 17.55–21.70 kg ha^-1^ P_2_O_5_, and 53.23–67.29 kg ha^-1^ K_2_O. The optimal fertilization measures to achieve > 23.41 pods per plant intersected with those interval for yield, and the optimal N fertilization to achieve a 100-seed weight > 6.58 g intersected with the interval for yield; however, the optimal N, P, and K fertilization for seeds per pod did not. The appropriate N, P, and K application rates, therefore, should be determined from the optimal fertilization measures for yield and pods per plant., moreover, it should be considered the applications of N fertilizer on 100-seed weight.

Optimal fertilization measures, however, varied with variety, planting density, and soil conditions [26, 28, 32, 33]. Therefore, in mung bean production, all of the aforementioned parameters must be fully considered [34]. However, the most challenging aspect of N, P, and K application is optimizing its use efficacy. Most of the N- and P-based fertilizers commercially available in the agrochemical market have an use efficiency < 30% because of rapid volatilization into greenhouse gases or fixation with other elements. According to production practices and the results of our study, mung bean fertilizer application should reduce N, increase K, and stabilize P. By lowering the application of N fertilizer, rhizobia will be free to fix atmospheric N. K fertilizer doses should be regulated to optimize crop growth and development. P fertilizer application should be stabilized to improve its utilization by plants. With precise, optimized fertilization, the utilization of these nutrients by crop could be increased, which in turn improves crop fertilization efficacy, environmental protection, crop quality, and crop yield. Future studies should focus on the effects of N, P, and K fertilization on rhizobia, the regulation of N, P and K, and the optimization of fertilizer utilization and application rates.

## Conclusions

All three N, P, and K fertilizers significantly influenced the pods per plant and yield, which sharply increased and then gradually decreased with the increasing N, P, and K fertilizers. However, the 100-seed weight significantly increased with the increasing N fertilizer, and significantly decreased with increase in the P fertilizer, but the K fertilizer effect on 100-seed weight was non-significant. Moreover, the seeds per pod significantly decreased with the increasing N and K fertilizers, and had no significant change with the P fertilizer. The interaction effects of the three nutrients on yield and pods per plant were NP > NK > PK. The NP interaction had a significant effect on yield and pods per plant at high N levels, while the NK interaction had a significant effect on yield at low N levels. The optimal fertilization measures for a yields >2,141.69 kg ha^-1^ were 34.38–42.62 kg ha^-1^ N, 17.55–21.70 kg ha^-1^ P_2_O_5_, and 53.23–67.29 kg ha^-1^ K_2_O. The optimal N, P, and K fertilization interval to achieve a pods per plant > 23.41 intersected with the interval for yield, and the optimal fertilization interval for N fertilizer to achieve a 100-seed weight > 6.58 g intersected with the interval for yield, but the seeds per pod did not. This may be due to the seeds per pod could maintain a stable value within the certain levels of N, P, and K fertilizers, while it maybe affected more by the genotypes at such situations; these problems should be further investigate in the future. The maximum yield was 2,394.60 kg ha^-1^ at 41.9 kg ha^-1^ N, 20.7 kg ha^-1^ P_2_O_5_, and 66.50 kg ha^-1^ K_2_O. The best fertilizer ratio was N:P_2_O_5_:K_2_O = 1:0.5:1.59. Yield did increased by using the optimal fertilization measures compared to that obtained using conventional fertilization during the validation test. To sum up, the reasonable optimization of N, P, and K fertilization could achieve high mung bean yield, improve yield components, and obtain products of full and large size grain. In the production practice, we should determine the optimal fertilization scheme according to the soil fertility condition, and refer to the optimal N, P, and K fertilizer rate in this study, with the principles of reducing N, stabilizing P and increasing K.

## Supporting information

S1 Fig Meteorological data of Baicheng city, jilin province, China, from 2013 to 2015. Average temperature (a), Rainfall (b), Sunshine duration(c).
(TIF)
