# Supplementary figures and images for: Nitrogen, phosphorus, and potassium fertilization to achieve expected yield and improve yield components of mung bean
Source: PLoS One. 2018 Oct 25;13(10):e0206285. doi: 10.1371/journal.pone.0206285 (PMC6201933; doi:10.1371/journal.pone.0206285)

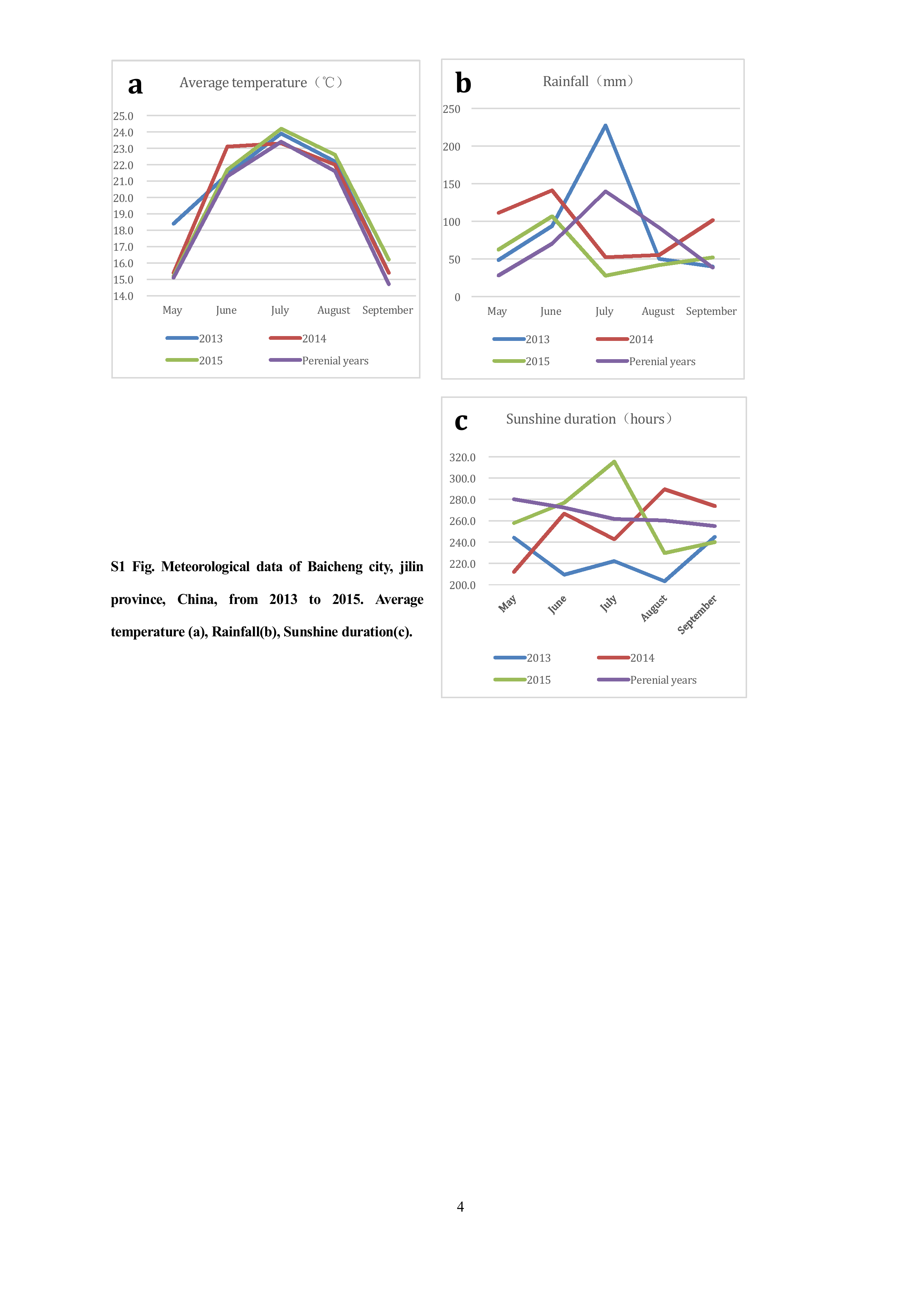

Supplement: S1 Fig — Average temperature (a), Rainfall (b), Sunshine duration(c). (TIF) [file pone.0206285.s001.tif]
